# Lactylation of the SARS-CoV-2 spike protein is required for viral infection

**DOI:** 10.1038/s41392-025-02428-z

**Published:** 2025-10-06

**Authors:** Jingguo Xin, Chunlei Wang, Zhaolong Li, Wenying Gao, Wenyan Zhang

**Affiliations:** Center of Infectious Diseases and Pathogen Biology, Institute of Virology and AIDS Research, Key Laboratory of Organ Regeneration and Transplantation of The Ministry of Education, The First Hospital of Jilin University, Changchun, China

**Keywords:** Biochemistry, Cell biology

Dear Editor,

The primary mechanism by which SARS-CoV-2 invades the host is the binding of the spike (S) protein to the angiotensin-converting enzyme 2 (ACE2) receptor and subsequent membrane fusion.^1^ During the cell entry process, SARS-CoV-2 S protein is cleaved into the S1 (responsible for ACE2 binding) and S2 (anchoring the S protein to the membrane and mediating membrane fusion) subunits by cellular proteases. Additionally, palmitoylation of the SARS-CoV-2 S protein enhances the binding affinity of the virus for the ACE2 receptor, thereby increasing viral infectivity.^2^ However, whether the SARS-CoV-2 S protein undergoes other posttranslational modifications (PTMs) that regulate S protein function remains unclear.

Protein lactylation was initially identified as occurring on histone lysine residues in 2019,^3^ which has been gradually observed on nonhistone proteins as well.^4^ Recent studies have reported that lactylation of host proteins during viral infection affects virus replication and reactivation.^4^ Although SARS-CoV-2 infection leads to intracellular lactic acid accumulation and muscle soreness, whether SARS-CoV-2-encoded viral proteins undergo lactylation and their role and regulatory mechanisms in SARS-CoV-2 infection have not been reported.

To determine whether increased lactate levels are closely associated with SARS-CoV-2-encoded viral proteins, we examined the lactylation levels of four structural proteins and found that the S protein underwent lactylation (Fig. 1a). Furthermore, exogenous lactate (sodium lactate, NaLa) treatment promoted the S lactylation level, whereas treatment with 2-DG, a glycolysis inhibitor that reduces the intracellular lactate concentration, or oxamate, a pyruvate analog that competitively inhibits the activity of lactate dehydrogenase (LDH), reduced the S lactylation level (Fig. 1a). These findings confirm that the S protein is subject to lactylation. The specific lactylation sites K424, K776, and K1028 on the S protein were subsequently identified through mass spectrometry (MS) analysis. By immunoprecipitation (IP) experiments, we confirmed that, compared with the wild-type S protein (S-WT), the S-K424R, S-K776R, and S-K1028R mutants presented reduced lactylation levels, with a cumulative effect observed when all three sites were simultaneously mutated (Fig. 1a).

**Fig. 1:**
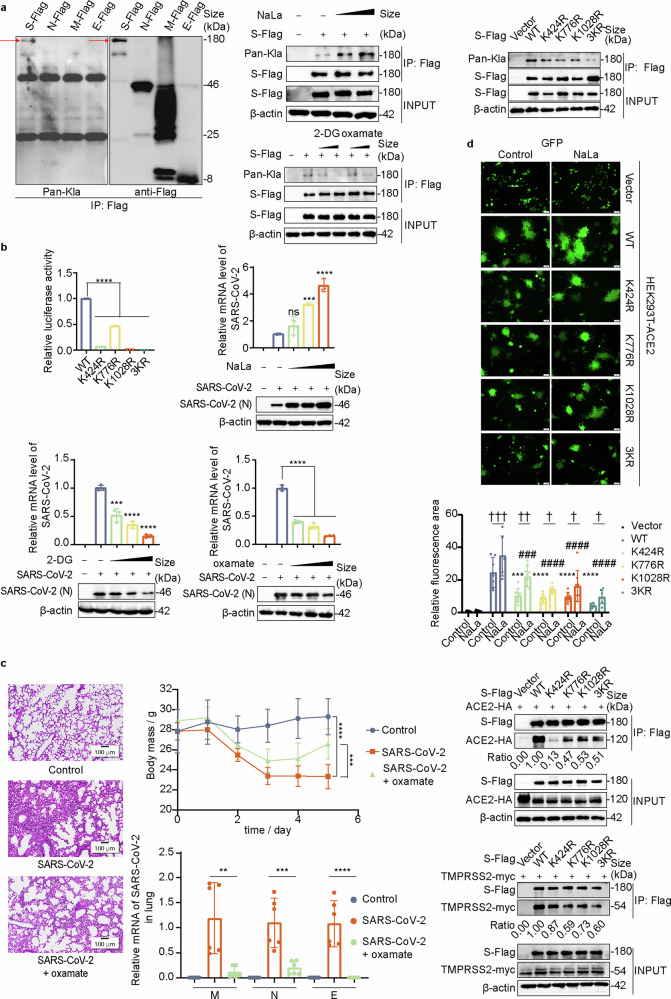
Lactylation of the S protein promotes viral infection. **a** Lactylation occurs on the S protein of SARS-CoV-2. S/N/M/E-Flag plasmids were transfected into HEK293T cells for 48 h, after which the cells were harvested, and the lysates were coincubated with protein G-agarose and Flag antibodies to enrich the structural proteins. Protein lactylation was probed with a Pan-Kla antibody via western blot (WB). Exogenous lactate treatment increases the lactylation level of the S protein. S-Flag was transfected into HEK293T cells, and the cells were then treated with NaLa (25 mM, 50 mM) 12 h before harvest. At 48 h posttransfection, the cells were collected for IP analysis to examine the lactylation level of the S protein. 2-DG or oxamate treatment inhibits S protein lactylation. S-Flag was transfected into HEK293T cells for 36 h, after which the cells were treated with 2-DG (5 mM, 10 mM) or oxamate (10 mM, 20 mM) for another 12 h before harvest. S-WT/K424R/K776R/K1028R/3KR-Flag were transfected into HEK293T cells for 48 h, after which lactylation levels were detected. **b** S lactylation is essential for the infectivity of SARS-CoV-2 pseudoviruses. HEK293T-ACE2 cells were infected with S-WT or mutant pseudoviruses for 72 h. Infection efficacy was analyzed by measuring firefly luciferase activity relative to the S-WT level (set as 1) (*n* = 3). Statistical analysis was performed via one-way ANOVA. Exogenous NaLa treatment promoted SARS-CoV-2 infectivity, whereas 2-DG or oxamate treatment inhibits SARS-CoV-2 infectivity. HEK293T-ACE2 cells were infected with the SARS-CoV-2 BA.5 variant and then treated with NaLa (10 mM, 25 mM, or 50 mM), 2-DG (2 mM, 5 mM, or 10 mM) or oxamate (5 mM, 10 mM, or 20 mM). N protein expression levels in cell lysates and N mRNA levels in the cell supernatant were assayed 48 h later. **c** Representative images of H&E-stained lungs from differently treated mice. (scale bar, 100 μm). Weights of the mice monitored over the experimental duration. Viral RNA loads in mouse lungs were detected at 7 dpi by measuring the mRNA levels of M, N and E. **d** S-lactylation is crucial for S-mediated membrane fusion. HEK293T cells coexpressing S-WT/K424R/K776R/K1028R/3KR-Flag and GFP were cocultured with HEK293T-ACE2 cells. Cell fusion was measured by fluorescence microscopy after 24 h (scale bar, 50 μm). The fusion areas were quantitatively analyzed via ImageJ software, and statistical analysis was performed via two-way ANOVA (* for comparisons versus the control group-WT; # for comparisons versus the NaLa group-WT; and † for comparisons between selected groups). S mutants show reduced binding to ACE2 and TMPRSS2. ACE2-HA or TMPRSS2-myc were transfected into HEK293T cells along with S-WT-Flag or its mutants for 48 h, after which protein interactions were detected by co-IP. (All subfigures follow the same statistical criteria. ns, not significant, *p < 0.05 (#, †), **p < 0.01 (#, †), ***p < 0.001 (#, †), ****p < 0.0001 (#, †))

To investigate whether lactylation of the S protein affects SARS-CoV-2 infection, luciferase-expressing pseudoviruses bearing either S-WT or S mutants (S-K424R, S-K776R, S-K1028R, or S-3KR) were generated in an envelope-defective HIV-1 backbone. The entry efficiency of these pseudoviruses was examined by analyzing luciferase activity in transfected HEK293T-ACE2 cells. Compared with the S-WT pseudovirus, which was normalized by quantitative PCR, the S protein mutant pseudovirus presented a reduction in luciferase activity (Fig. 1b), indicating that the entry of SARS-CoV-2 pseudoviruses is highly dependent on the S lactylation level. We further infected HEK293T-ACE2 cells with the SARS-CoV-2 BA.5 variant to examine the effect of the S lactylation level on virus replication. NaLa treatment also promoted SARS-CoV-2 replication (Fig. 1b), whereas lactylation inhibitors (2-DG and oxamate) had the opposite effect (Fig. 1b).

To further investigate the critical role of lactylation in vivo, BalB/c mice were treated with oxamate and subsequently infected with a mouse-adapted SARS-CoV-2 strain. Oxamate treatment significantly reversed weight loss and reduced the viral load in the lungs (Fig. 1c), accompanied by substantial alleviation of pulmonary injury (Fig. 1c). These findings collectively indicate that inhibition of S protein lactylation attenuates SARS-CoV-2 infectivity.

We next investigated the mechanism by which S-lactylation affects SARS-CoV-2 infection. S-mediated membrane fusion is critical for virus entry. Therefore, we investigated whether S-lactylation affects membrane fusion efficacy. Fluorescence imaging revealed that S-WT mediated cell‒cell fusion well, whereas S mutants presented a significant reduction in the fusion area. Furthermore, NaLa treatment enhanced the fusion capacity of S-WT but had little effect on the lactylation-deficient mutants (Fig. 1d).

Since the S1 subunit of the S protein is responsible for ACE2 binding, we examined the binding ability of S-lactylation-defective mutants to ACE2. Compared with the S-WT protein, three mutants presented weaker binding to ACE2, especially K424R (located in the S1 subunit) (Fig. 1d). These findings suggest that lactylation may influence S and ACE2 interactions by neutralizing key charges, altering the RBD conformation. We also examined the interaction of S mutants with the transmembrane protease serine 2 (TMPRSS2), which is responsible for S2 site cleavage to expose the fusion peptide and promote membrane fusion. The results showed that the S protein efficiently interacted with TMPRSS2, while its mutants, especially K776R and K1028R (located in the S2 subunit), had weakened binding to TMPRSS2 (Fig. 1d). Overall, S protein lactylation is crucial for ACE2 and TMPRSS2 binding.

In summary, we identified lactylation as a previously unrecognized PTM occurring on the SARS-CoV-2 S protein that enhances S protein interaction with the host receptor ACE2 or the host protease TMPRSS2, thereby promoting viral entry and infection. However, our MS data and recent studies ^5^ revealed that multiple PTMs occur on the S protein, including coexisting lactylation and acetylation at K424, K776, and K1028. Therefore, cross-talk between different PTMs may exist, and a potential role for acetylation cannot be completely excluded. This study highlights the important role of lactylation in SARS-CoV-2 infection and provides new insights for the development of antiviral strategies. Further research is needed to identify the specific enzymes responsible for lactylation, which may help elucidate the regulatory networks involving lactylation and other PTMs in the modulation of S protein function.

## Supplementary information

Sigtrans_Supplementary_Materials

Dataset 1

Dataset 2

## Data Availability

The authors declare that all the data supporting the findings of this study are available within the paper and its supplementary information files.
