# Supplementary material for: Lactylation of the SARS-CoV-2 spike protein is required for viral infection
Source: Signal Transduct Target Ther. 2025 Oct 6;10:329. doi: 10.1038/s41392-025-02428-z (PMC12497866; doi:10.1038/s41392-025-02428-z)
Supplement: Supplementary file 1 — Sigtrans_Supplementary_Materials [file 41392_2025_2428_MOESM1_ESM.docx]

Supplementary Materials for

Lactylation of SARS-CoV-2 Spike protein is required for viral infection

Jingguo Xin^1^, Chunlei Wang^1^, Zhaolong Li^1^, Wenying Gao^1, *^, Wenyan Zhang^1, *^

^1^Center of Infectious Diseases and Pathogen Biology, Institute of Virology and AIDS Research, Key Laboratory of Organ Regeneration and Transplantation of The Ministry of Education, The First Hospital of Jilin University, Changchun, China.

Correspondence to: Wenyan Zhang (zhangwenyan@jlu.edu.cn), Wenying Gao (gaowenying@jlu.edu.cn)

**This file includes:**

Supplementary materials and methods

**Materials and Methods**

***Cells and Plasmids***

HEK293T (American Type Culture Collection [ATCC], Manassas, VA, USA, catalog no. CRL-11268) and HEK293T-ACE2 (laboratory preparation) cells were cultured as monolayers in Dulbecco’s modified Eagle’s medium (DMEM) (Hyclone, Logan, UT, USA, catalog no. 11965092), which was supplemented with 10% heat-inactivated (56℃, 30 min) fetal bovine serum (FBS, PAN-Biotech, Adenbach, GER, catalog no. ST30-3302), and maintained at 37℃ with 5% CO_2_ in a humidified atmosphere.

S-Flag (codon optimized in accordance with the human genome) carrying a Flag tag inserted into pCAG was a gift of Professor Wang Peihui (Shandong University). Mutants of S-Flag were constructed by the Comate Bioscience company (Changchun, CHN). ACE2-HA and myc- TMPRSS2 were purchased from Miaoling Biotechnology (Wuhan, CHN, catalog no. P46385 and P5570).

***Transfection***

DNA transfections were carried out by PEI Reagent (Polyscience, Warrington, USA, catalog no. 23966-1) according to the manufacturer’s instructions.

***Cell-cell fusion assays***

Flag-S and pEGFP-N1 plasmids at 5:1 ratio were co-transfected into HEK293T effector cells. HEK293T-ACE2 cells were used as target cells. 5×10^4^ target cells (HEK293T-ACE2) were incubated in 24-well plates at 37℃ for 4 h, followed by the addition of 1×10^5^ HEK293T effector cells. After coculture for 24 h, syncytium formation was observed under the inverted fluorescence microscope.

***Production and infection of SARS-CoV-2 S pseudoviruses***

To generate the SARS CoV-2 S pseudotyped HIV-1 single-round luciferase virus, 4×10^6^ HEK293T cells were co-transfected with pLP1, pLP2, pCDH-CMV-Luciferase-CopGFP and pCAG-SARS-CoV-2-S (WT and mutants) using the PEI transfection reagent according to the manufacturer’s instructions. The cells were transferred to fresh DMEM 6 h later. 48 h later, the supernatant containing SARS-CoV-2 pseudoviruses were harvested, and then centrifuged at 8000 rpm for 5 min to pellet any cell debris and filtered through a 0.45 μm filter. 200 μL viral supernatant was used to extract viral RNA and digested with DNase Ⅰ to eliminate plasmid contamination, and viral RNA copies were detected by RT-qPCR using forward primer: 5’-GGCACTGACAATTCCGTGGT-3’, reverse primer: 5’-AGGGACGTAGCAGAAGGACG-3’. Then, HEK293T-ACE2 cells were seeded into 24-well plates and infected with pseudotyped viruses at equal RNA copies supplemented with polybrene (4 mg/mL). After incubation for 12 h, the pseudovirus-containing supernatant was removed and replaced with fresh DMEM containing 10% FBS. After 72 h post-infection, the HEK293T-ACE2 cells were lysed with 50 μL passive lysis buffer (Promega, Madison, WI, USA, catalog no. E1910) to measure firefly luciferase activity using a luciferase reporter assay system (Promega, Madison, WI, USA, catalog no. E1910) according to the manufacturer’s protocol. Viral infection rate was calculated by analyzing the mean firefly luciferase activity as to S-WT group (set as 1).

***Viruses and virus infection***

SARS-CoV-2 BA.5 strain was propagated in Vero cells in DMEM supplemented with 2% FBS, and titer assayed by using the median tissue culture infectious dose (TCID_50_) assay. For SARS-CoV-2 infection, cells grown to 70% confluence in the 12-well plates were washed twice with phosphate-buffered saline (PBS) and incubated with SARS-CoV-2 isolate at 37°C for 2 h at a MOI of 0.01. Plates were gently agitated at 15 min intervals to facilitate adsorption. After adsorption, the virus-containing medium was replaced with fresh medium containing 2% FCS, followed by incubation at 37℃ in 5% CO_2_ for the indicated durations. All experiments of infectious SARS-CoV-2 were conducted under Biosafety Level 3 facilities.

***Western blot (WB) and Immunoprecipitation (IP)***

Transfected and infected HEK293T, or HEK293T-ACE2 cells were harvested and boiled in 1× loading buffer (0.08 M Tris, pH 6.8, with 2.0% SDS, 10% glycerol, 0.1 M dithiothreitol and 0.2% bromophenol blue) followed by separation on a 10% or 12.5% polyacrylamide gel. Proteins in the samples were transferred onto a polyvinylidene fluoride (PVDF) membrane for WB analysis. The membranes treated with sealer solution for 1 h were incubated with indicated primary antibodies, and followed by a corresponding horseradish peroxidase (HRP)-conjugated secondary antibody (Jackson Immunoresearch, West Grove, USA, catalog no. 115-035-062 for anti-mouse and 111-035-045 for anti-rabbit) diluted 1:10000 respectively. At last, the proteins on the PVDF membranes were visualized by the super sensitive ECL luminescence reagent (Meilunbio, Dalian, CHN, catalog no. MA0186).

The following antibodies were used in this study: SARS-CoV-2 nucleocapsid antibody (GeneTex, Irvine, CA, USA, catalog no. GTX635679), anti-L-Lactyl Lysine Rabbit mAb (PTMBio, Hangzhou, CHN, catalog no. PTM-1401RM), anti-myc pAb, (Proteintech, catalog no. 16286-1-AP), anti-hemagglutinin (anti-HA) pAb (Invitrogen, Carlsbad, USA, catalog no. 71-5500), anti-β-actin monoclonal antibody (mAb) (GenScript, Nanjing, CHN, catalog no. A00702), anti-Flag mAb (Sigma, Saint Louis, USA, catalog no. F1804), anti-GFP mAb (Abcam, catalog no. ab1218).

For IP of proteins with a Flag tag, HEK293T cells transfected with indicated plasmids for 48 h, were harvested and washed twice with the cold PBS, followed by ultrasonication in a lysis buffer [PBS containing 1% Triton X-100 and complete protease inhibitor cocktail (Roche, Basel, Basel-City, SUI, catalog no. 11697498001)]. Cell lysates cleared by centrifugation at 12000 rpm for 30 min at 4℃ were mixed with protein-G agarose beads (Roche, catalog no. 11243233001) and anti-Flag monoclonal antibody and incubated at 4℃ for 4 h on an end-over-end rocker. The beads which bound proteins were then washed six times with the cold wash buffer (20 mM Tris-HCl, pH 7.5, 100 mM NaCl, 0.1 mM EDTA, 0.05% Tween-20). Proteins drop-down by beads were analyzed by WB.

***RNA extraction and Reverse transcription quantitative PCR (RT-qPCR)***

For RT-qPCR, intracellular and viral RNA was extracted from cell lines or cell supernatant used in this study with Trizol reagent (Invitrogen, Carlsbad, USA, catalog no. 15596026CN) and diethyl pyrocarbonate (DEPC)-treated water. The cDNAs were generated by a MonScript™ RTIII All-in-One Mix kit (Monad Biotech Company, Suzhou, CHN, catalog no. MR05001S/M) according to the manufacturer’s instructions. Reverse transcription was carried out in a 20 μL volume, which contained 1 μg RNA extracted from the above samples. qPCR was carried out by the SuperStar Iniversal SYBR Master Mix (CWBio, Taizhou, CHN, catalog no. CW3360M). The qPCR assay was carried out in a 20 μL volume consisting of 1 μL of 5 μmol/L of each oligonucleotide primer, 2 μg of cDNA templates, and 10 μL of SYBR Master Mix. Amplifications of the target fragment were carried out as the following steps: initial activation of the Taq DNA Polymerase at 95℃ for 2 min, and then followed with 40 cycles of 95℃ for 15 s, 57℃ for 15 s and 68℃ for 20 s. The primers used in this study: N-SARS-CoV-2-RT-F: 5’-GGGGAACTTCTCCTGCTAGAAT-3’, N-SARS-CoV-2-RT-R: 5’-CAGACATTTTGCTCTCAAGCTG-3’, 5’-E-SARS-CoV-2-RT-F: CGATCTCTTGTAGATCTGTTCTC-3’, E-SARS-CoV-2-RT-R: 5’-ATATTGCATTGCAGCAGTACGCACA-3’, M-SARS-CoV-2-RT-F: 5’-GGTTTCCTATTCCTTACATGG-3’, M-SARS-CoV-2-RT-R: 5’-ATTCTGTAAACAGCAGCAAGC-3’.

***Mouse lines and infection***

BALB/c mice were purchased from Changsheng biotechnology company (Liaoning, CHN). All welfare and experimental procedures were carried out strictly in accordance with the Guide for the Care and Use of Laboratory Animals and the related ethical regulations. All efforts were made to minimize animal suffering. The mice were randomly divided into three groups, and each group contained six mice. One group was treated with the dosage of 300 mg/kg oxamate for five times every two days via intraperitoneal injection, then infected with a mouse-adapted SARS-CoV-2 variant (GenBank: OL913104.1) at a dosage of 10^5.5 TCID_50_/ml via intranasal challenge. No oxamate treatment and SARS-CoV-2 infection were used as a negative control.

***Statistical analysis***

The detailed statistical analysis has been described in figure legends. All data are expressed as the mean ± standard deviations (SDs). Statistical comparisons were made using One-Way ANOVA or Two-Way ANOVA according to different situations.
