# Supplementary material for: Lactylation of the SARS-CoV-2 spike protein is required for viral infection
Source: Signal Transduct Target Ther. 2025 Oct 6;10:329. doi: 10.1038/s41392-025-02428-z (PMC12497866; doi:10.1038/s41392-025-02428-z)
Supplement: Supplementary file 2 — Dataset 1 [file 41392_2025_2428_MOESM2_ESM.pdf]

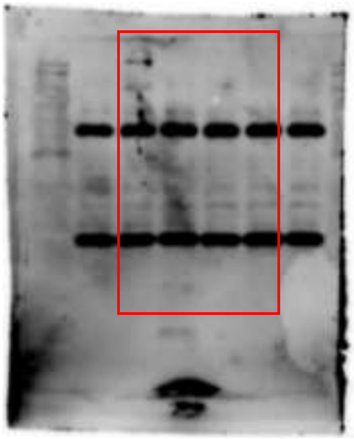

Fig. 1a Pan-Kla

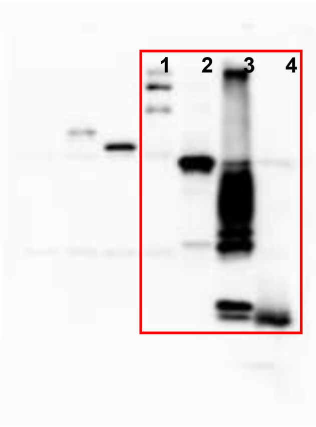

Fig. 1a anti-Flag

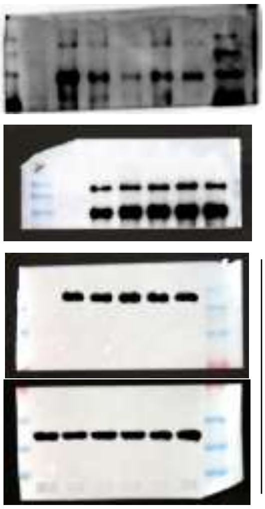

Fig. 1a IP-Pan-Kla

Fig. 1a IP-S-Flag

Fig. 1a INPUT-S-Flag

Fig. 1a INPUT-β-actin

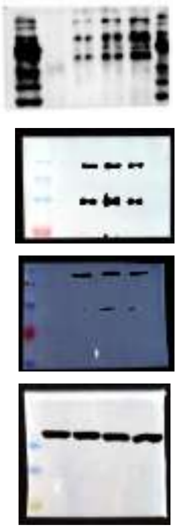

Fig. 1a IP- Pan-Kla

Fig. 1a IP-S-Flag

Fig. 1a INPUT-S-Flag

Fig. 1a INPUT-β-actin

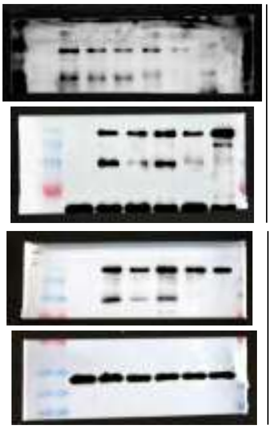

Fig. 1a IP-Pan-Kla

Fig. 1a IP-S-Flag

Fig. 1a INPUT-S-Flag

Fig. 1a INPUT-β-actin

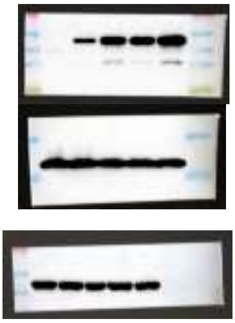

Fig. 1b N

Fig. 1b Histone (not displayed)

Fig. 1b β-actin

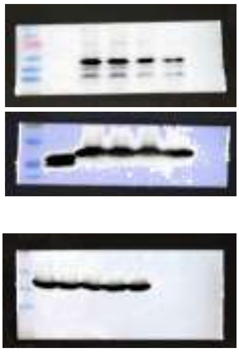

Fig. 1b N

Fig. 1b Histone (not displayed)

Fig. 1b β-actin

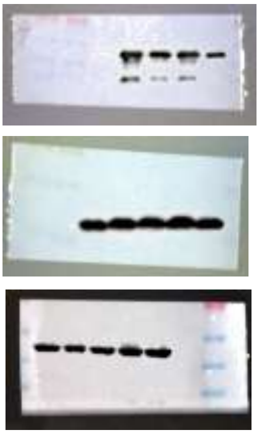

Fig. 1b N

Fig. 1b Histone (not displayed)

Fig. 1b β-actin

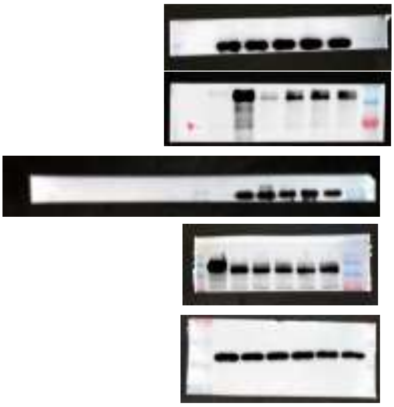

Fig. 1d IP-S-Flag

Fig. 1d IP-ACE2-HA

Fig. 1d INPUT-S-Flag

Fig. 1d INPUT-ACE2-HA

Fig. 1d INPUT-β-actin

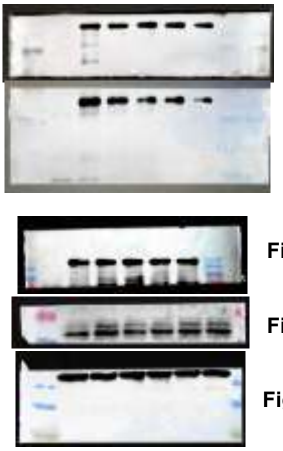

Fig. 1d IP-S-Flag

Fig. 1d IP-TMPRSS2-myc

Fig. 1d INPUT-S-Flag

Fig. 1d INPUT-TMPRSS2-myc

Fig. 1d INPUT-β-actin
